# Oral Health, Antimicrobials and Care for Patients With Chronic Oral Diseases – A Review of Knowledge and Treatment Strategies

**DOI:** 10.3389/froh.2022.866695

**Published:** 2022-06-07

**Authors:** Mark Ide, Malika Karimova, Jane Setterfield

**Affiliations:** Centre for Host-Microbiome Interactions, Faculty of Dentistry, Oral and Craniofacial Sciences, King's College London, London, United Kingdom

**Keywords:** oral health, periodontitis, gingivitis, microbiome, pemphigus vulgaris, lichen planus, mucous membrane pemphigoid, mucosal disease

## Abstract

Periodontal and chronic oral mucosal diseases are significant life impacting conditions which may co-exist and synergistically act to cause more severe and widespread oral pathology with enhanced challenges in effective management. Clinicians regularly observe these effects and struggle to effectively manage both problems in many patients. There is limited understanding of many basic and applied scientific elements underpinning potentially shared aetiopathological features and management. Recent developments in translational science provide an opportunity to greater improve knowledge and subsequently care for patients with these problems.

## Introduction

Periodontal diseases and chronic oral mucosal diseases (OMD) both have an impact on comfort, function, and quality of life [1–11]. Beyond this they may co-exist in some patients and although there is limited knowledge of the interactions between these conditions, there are good reasons to believe that they may act in a bidirectional manner to both exacerbate their severity and impact and to complicate management.

### Periodontal Diseases and Management

Periodontal diseases are processes driven by the patient's inflammatory response to the presence and accumulation of bacterial plaque and form a major part of the global heath burden [12–15]. They are known to result in significant impacts on function, wellbeing, other disease states and Quality of Life for the majority of populations in many states. Management of these problems is generally simple and based around control of local and systemic risk factors, with good home care the cornerstone for success, and supportive professional care where appropriate [16]. Outcomes from treatment are further enhanced by management of associated secondary risk factors including aspects of health and behavioral factors such as control of diabetes, smoking (and possibly vaping) cessation and, where appropriate, modification of other drug prescriptions to eliminate side effects [17].

Periodontal diseases can be subcategorised based on the nature and degree of changes seen and damage caused. The majority of disease falls largely into one of two groups [18], namely gingivitis and periodontitis.

Gingivitis is a condition in which there is inflammation of the marginal soft tissues around the neck of the tooth. It is completely reversible but is also extremely common. There are associated shifts in the local microflora in the presence of gingivitis [19–21]. There is no irreversible damage to supporting tissues, however, it is possible for this condition to subsequently progress to periodontitis [22]. Gingivitis can be controlled by simple home care measures, supported by professional interventions at an early phase with occasional review [23, 24].

Periodontitis is a more advanced condition in which there is (frequently) irreversible destruction of supporting soft and hard tissues around teeth. This can lead to mobility, acute infection, pain and ultimately tooth loss. Again there are recognized shifts in microflora in periodontitis, which may be further affected by recognized systemic factors [25]. Periodontitis management is primarily focussed on stopping progression, with clinically significant regeneration of lost tissues only being predictable in an increasing but still limited number of scenarios [16, 26].

Although periodontitis may be expected in all adult humans in the absence of oral hygiene, the onset, extent and rate of development is known to vary between individuals [27, 28], and this is equally true even if “normal” oral hygiene is practiced [29]. Patients with particularly higher levels of susceptibility to periodontitis are required to deliver a very high standard of plaque control supported by regular professional maintenance in order to prevent further ongoing inflammation and tissue damage [30–33]. However in the presence of good maintenance, even questionable teeth can be maintained for a long period of time [30, 34–37].

Likewise, regeneration of tissues is presently only feasible in patients with exemplary oral hygiene where other risk factors have also been managed [16, 38, 39].

### Oral Mucosal Conditions

Oral mucosal diseases form a group of chronic predominantly immune mediated disease, such as lichen planus (OLP), pemphigus vulgaris (PV), bullous pemphigoid (BP), dermatitis herpetiformis, Epidermolysis bullosa acquisita (EBA), mucous membrane pemphigoid and linear IgA bullous disease [40]. The pathogeneses of these diseases are not completely clear, but autoantibodies play critical roles in their pathogenesis [41–43]. The majority of these diseases are rare. Clinically they are characterized by formation of blisters and erosions in oral mucosa, often presenting as desquamative gingivitis. Erosive gingival lesions can be the source for the microbial accumulation and may hence trigger periodontal tissue breakdown [44]. They often clinically overlap with each other; therefore, immunological and histopathologic tests are requested for differential diagnosis.

### The Microbiome and Aspects of Periodontal Disease and Management

The role of plaque as an aetiological factor in, and its removal in management of, periodontal diseases has been known for hundreds of years. However, in the last 40 years with the advent of new technologies our understanding the role of the oral microflora has expanded significantly. Regardless there are still large gaps in our knowledge, such that although we understand which simple measures are likely to work in many patients, it is still not fully clear why, or why their efficacy varies between individuals. Individual variation in disease susceptibility and rate of disease progression has been attributed to genetic or epigenetic variations in host inflammatory response, including neutrophil function, cytokine production and macrophage activity [45–50], to specifical microbial infection (such as with clones of *Aggregatibacter actinomycetemcomitans*) [51] or to a combination of these, whereby one leads to the other with debate around which happens first. Current concepts of the microbiology of periodontal disease, and more specifically periodontitis, relate to the presence of groups of putative periodontal pathogens described as “complexes” (based on their pathogenic potential, association with each and presence in diseased sites). These concepts first arose with the advent of early nonculture-based means of microbial assessment such as “checkerboard” DNA-DNA hybridisation [52, 53] and have been supplemented and superseded by more sophisticated methods such as qt-PCR [54] and high throughput gene sequencing and complex analysis [55]. Supplemented by metabolomic analysis and other approaches, a massive volume of potential information is available to better understand the pathobiology of both periodontal and mucosal diseases.

More recently the concept of dysbiosis induced by various environmental shifts and “keystone pathogens” has gained support [56–58]. However, this does not fully explain all aspects of what is without doubt a complex series of interactions between the environment created by the host (and potential variations) combined with those within the microbiome [59, 60]. This may lead to patient or even site-specific disruptions responsible for disease activation of progression, with no universal pattern seen across all hosts [61]. It has also been suggested that such modified dysbiotic microbiota can form stable communities that may additionally be suppressed but not eliminated by antimicrobial therapies [62]. There is an extensive literature confirming that regardless of OMD status, development and maturation of dental plaque can potentiate local inflammatory change and hence likely periodontal disease progression [63–65].

This picture is further muddied by the complex nature of the oral microbiota [66], and the belief that many microorganisms present have not yet been formally identified or characterized. In addition, the ability to transfer resistance genes and evade antimicrobials of organisms within a plaque biofilm also is likely to impact on future strategies for patient management especially if these agents are used for other purposes [67].

A further central aspect of periodontitis management relates to modifiable risk factors that further influence susceptibility to disease by changing individual host responses. These include smoking and possibly vaping, uncontrolled diabetes and possibly obesity, medications that can change cellular activity in gingival tissues or drugs which result in oral dryness or xerostomia [16, 68]. Changes in systemic health, lifestyle or medications, or variation in host response can therefore relate to and influence the oral microbiome, leading to specific individual patterns [69].

Current consensus for the management of periodontal diseases is based on a foundation of effective daily self-performed plaque removal and/or biofilm disruption combined with professional interventions to complement or facilitate this [68]. The effectiveness of this essentially non-specific approach has been confirmed by national and international guidelines for care [16, 70–73].

Whilst chemical means of plaque control are supported in certain circumstances, they are not seen as a mainstay of longer-term therapy for the majority of patients with periodontitis due to either limited efficacy and / or the presence of a range of undesirable complications. Even so it has been established that use of common means of chemical plaque control such as chlorhexidine used topically as a mouthwash were associated with further large shifts in microbiome biomass as well as diversity [20, 21, 69, 74].

### The Microbiome and Aspects of Chronic Oral Mucosal Disease

The microbiome of oral mucosal diseases has been investigated for the last couple of decades [75]. Few studies focus on lichen planus, but one describes the increase of periodontal pathogens such as *Porphyromonas gingivalis, Aggregatibacter actinomycetemcomitans, Treponema denticola, Prevotella intermedia* and *Tannerella forsythia* in subgingival plaque of OLP patients [76], and another describes changes in both richness and diversity of subgingival plaque associated with the presence of OLP when co-existing with periodontitis [77]. The role of the microbiome in autoimmune bullous disease (AIBD) is less investigated. One study on skin microbiome of PV and BP patients did not show significant differences in a-and b-diversity in comparison with healthy controls [78]. However, another study of the gut microbiome of PV patients showed increases in *Flavonifractor* genera, which was correlated with increased levels of chronic inflammatory cytokines. It supported the concept that the dysbiosis in the gut may trigger inflammatory processes and cause the development of disease [79]. This corresponds to research suggesting that initially healthy mice showed a reduction in local microbial richness after immunization with EBA antibodies [80]. However, limitations of all current studies include low patient numbers due to the relative rarity of these diseases, supporting the need to investigate these links further.

### Interactions Between Periodontal Diseases and Chronic Oral Mucosal Conditions – Bidirectional?

Soft tissue tenderness, ulcerations and sometimes scarring associated with many OMD cause many patients to struggle to achieve good mechanical oral hygiene. This then leads to more local plaque formation and increased levels of marginal inflammation. This can manifest as localized gingivitis but may also predispose to acceleration of gingival recession, periodontitis and ultimately tooth loss in those who are more susceptible to this condition [44, 81–84].

Whilst we do not yet fully understand the true relationship, it appears feasible that the presence of active periodontitis and associated microorganisms could potentially exacerbate local mucosal disease. This could occur by locally enhanced inflammation and/or modulation of autoimmune responses, either alone [85] or facilitated by superinfection of mucosal lesions by the proteolytic organisms often identified in periodontally involved sites [86, 87]. These organisms can act to directly or indirectly promote local inflammatory damage and hence could increase the severity and duration of mucosal disease [86–89].

### Management of Periodontal Problems for Patents With Mucosal Disease

Hopefully, adequate management of OMD would reduce the likelihood of periodontal complications for those with OMD. However patients may find themselves in a situation where the co-existence of these conditions may be almost synergistic.

Systematic reviews such as that by Figuero et al. [90] suggest that antiseptic agents including essential oils, chlorhexidine, stannous fluoride, cetylpyridinium chloride, sanguinarine, sodium hexametaphosphate and delmopinol, delivered via toothpastes or mouthwashes, can effectively reduce plaque and gingivitis levels and may as a result offer benefits for tissues in patients with gingivitis or periodontitis. However, these are still recommended as an adjunctive therapy [16] supporting mechanical oral hygiene. Mucosal diseases present challenges in oral hygiene and plaque control in regions with ulceration due to discomfort caused not only by physical brushing but also potentially by toothpaste constituents. It has been suggested that conventional formulations containing certain detergents may act to exacerbate some ulcerative conditions intraorally [91–95]. Unfortunately, chemical methods of plaque are often also not helpful. Those agents widely recognized as having clinical efficacy in management of periodontal diseases are often not well tolerated by patients, due to discomfort and pain potentially caused by various constituents such as flavorings, astringents, essential oils and preservatives. Even if diluted to the lowest effective concentration, many patients still report significant pain. Consequently, many patients will resort to using non-foaming, detergent-free agents and softer brushes, although removal of detergent may not impair plaque control [94, 95].

As a result, it is important to consider management of mucosal inflammation and lesion resolution whilst also trying to simultaneously improve plaque control. However, this can create challenges in compliance for the delivery of active agents by patients as well as risks of drug interactions. A better understanding of the exact relationships between these conditions and their management strategies may be expected to allow development of more effective approaches to therapy.

### Local Agents for Periodontal or OMD Management

Topical steroids are widely used in treatment of all clinical symptoms of OMD, with a prime aim of reducing pain and inflammation. These are generally applied topically, and include 0.1% triamcinolone acetonide in orabase, 0.05% fluocinolone acetonide, 0.05% clobetasol propionate, or 0.05% halobetasol, A further approach has been intralesional injection of triamcinolone acetonide (20 μg/L) [96]. They often used as a first line drug with rare side effects and low cost [97].

Whilst topically applied agents have been proposed for periodontal management, these have largely been professionally applied, and have been shown to be of questionable efficacy and correspondingly low cost-effectiveness [98, 99]. The lack of high-quality evidence and significant clear clinical benefit has led to no clear recommendation for their use initial or longer term maintenance periodontal therapy [16]. There are no data related to the value of specific agents designed for periodontal management (such as topical slow release antimicrobial agents) in OMD management.

### Systemic Agents for Periodontal or OMD Management

Various systemic adjuncts have been proposed for periodontal management [100, 101], and these have aimed to either act as antimicrobials or to modulate host inflammatory responses, or both. There are limited data from RCTs comparing antimicrobial regimes, but recent network meta-analyses and reviews have suggested that some agents (primarily a combination of amoxicillin and metronidazole) when used at the end of a course of professional mechanical intervention can give enhanced clinical benefit in certain patient groups [100, 102], and this has led to guidelines and recommendations for clinical practice [16, 103] related to dosage, duration, timing and patient based criteria for use. Systemic antimicrobials are generally not considered a valid sole therapy for periodontal therapy in the absence of local biofilm control, a concept supported by *in vivo* studies [62].

Systemic agents are widely used in management of oral mucosal disease. Systemic steroids being used for AIBD because of its immunosuppressive effect to inhibit cytokines, which further prevents T cell activation [43, 104].

Azathioprine and mycophenolate mofetil are another two immunosuppressive drugs which reduce the number of circulating B and T lymphocytes, resulting in decreased immunoglobulin production and reduced IL-2 secretion, as well as inhibit the generation of cytotoxic T cells [105].

Rituximab is a biological agent directed against the B-lymphocyte-specific antigen CD20 which use where the treatment with other systemic agents such as those above has failed.

Even though immunosuppressive treatment is the first line treatment for autoimmune conditions, systemic antibiotics are an alternative treatment for oral mucosal disease. The positive effect of antibiotics can be explained by possible shared pathogenic mechanisms. The cross-talk activation of autoreactive B and T cells is explained by the mechanism of epitope spreading or molecular mimicry, may lead to immune cells are attacking host tissue, confusing them with the infection epitopes. One such antibiotic is dapsone, a sulfone derived antimicrobial agent with anti-inflammatory effects. This is often used as an adjuvant therapy for AIBD, and as a first line medication for LABD and dermatitis herpetiformis [106]. Tetracyclines have been used in AIBD for their anti-inflammatory action [107, 108]. A recent study supported the hypothesis that oral doxycycline is as effective as oral prednisolone and that doxycycline is less likely to cause severe side effects [109]. Tetracyclines were shown to inhibit the activity of antiphospholipase, scavenge free radicals, and inhibit various matrix metalloproteinases, as well as impair lymphocyte activity [110].

Macrolides form another group of antibiotics described in the literature showing a positive effect on AIBD. Mensing and Krausse [111] reported a decrease of hospitalization days, as well as side effects of steroid use, while using erythromycin in combination with methylprednisolone. Later, numerous studies showed that erythromycin alone showed improvement in patients with bullous pemphigoid and linear IgA disease [112, 113].

However, some antibiotics are capable of triggering autoimmune mucosal disease. The literature describes the case of AIBD induced with ciprofloxacin, levofloxacin, and penicillin groups of antibiotics [114], but the exact mechanism and the overall impact compared to benefit unclear. It has also been suggested that thiol/phenol drugs may activate acantholytic enzymes or stimulate keratinocytes to release proinflammatory cytokines and trigger acantholysis through other mechanisms [115, 116].

### Implications for Self Care and for Professionally Delivered Care

All of these challenges mean that OMD patients often require personalized diagnosis, focussed and sometimes more frequent professional follow up and support for effective primary and secondary prevention of periodontal disease, supported by OMD management. This would ideally be delivered by dental hygienists or therapists, although suitably trained oral health educators may have a role [117, 118].

However, studies have shown consistently and conclusively that the main determinant for long term success in periodontal management is effective oral hygiene [23, 24, 119, 120]. Without this, treatments are known to deliver poorer clinical outcomes with corresponding microbiological changes.

As outlined above, this is presently hard to achieve for many patients, but initial studies (with limited patient numbers) are promising in this regard, even without the use of chemical adjuncts to therapy [88, 121–125].

Whilst it is generally accepted that the presence and management of local and systemic risk factors influences both initial periodontal therapy and the frequency of subsequent maintenance, the presence of OMD per se is not part of most routine paradigms. In fact, the literature focusses on local evidence such as inflammation and gingival bleeding, and the presence of residual periodontal pockets, and these have been shown to be effective and form the basis for current treatment protocols [126]. Hence when considering the frequency of professional recall, currently we are driven by the periodontal rather than other signs. It is unclear if this is suitable, based on the limited evidence base, for this group of patients.

### What Do We Need to Know?

Many patients with oral mucosal disease suffer significant problems ranging from discomfort and dietary restriction to tooth loss, loss of function and difficulty undergoing dental rehabilitation. This can occur in the presence or absence of periodontal disease, but where there is ongoing periodontitis and associated complications, management challenges and disease impacts are considerably greater. The most recent World Workshop Classification of Periodontal diseases [127] does not list OMDs as separate mucosal conditions that affect the periodontium, or as risk factors or grade modifiers in making a periodontitis diagnosis [128], and this may be worthy of review.

The advent of genomic approaches to characterize the oral and more specifically periodontal and lesional microbiome offers a novel and exciting way to understanding better not only whether these are linked, but also may allow better understanding of how these factors relate to the extent, severity and persistence of OMD. Beyond pathobiology, a knowledge of how these characteristics may change following the use of drugs or other interventions designed to manage periodontal diseases, OMD or both may allow more efficient care. We are only at the very beginning of this journey but there is much promise and much to be delivered. Topics worthy of further investigation include cross sectional studies informing our understanding of basic associations, and longer term work to better understand the impact of OMP management protocols on both the local and systemic host response, the lesional and oral microbiome and clinical manifestations of both conditions. This could be further enhanced by further development of disease classification to ensure that the epidemiological aspects of these problems can be better understood.

We are now aiming to deliver personalized, patient-centered care across medical and dental care. However, it is difficult to achieve a reasoned patient centered care pathway for OMD patients without having a better evidence-based understanding of the relationships as well as mechanisms underlying these, and the true nature of any bi-directional relationships, should they be found to exist. As a result there is a clear need for further basic and translational investigation work to better understand microbial and host-related aspects of the etiology and persistence of both conditions as well as related factors such as antimicrobial efficacy and resistance, in order to determine what treatments are most likely to work in which individuals [69]. This would be expected to offer the potential for major improvements in clinical outcomes and quality of life for this often overlooked but large body of patients.

## Author Contributions

All authors listed have made a substantial, direct, and intellectual contribution to the work and approved it for publication.

## Funding

This work was supported by internal funds.

## Conflict of Interest

The authors declare that the research was conducted in the absence of any commercial or financial relationships that could be construed as a potential conflict of interest.

## Publisher's Note

All claims expressed in this article are solely those of the authors and do not necessarily represent those of their affiliated organizations, or those of the publisher, the editors and the reviewers. Any product that may be evaluated in this article, or claim that may be made by its manufacturer, is not guaranteed or endorsed by the publisher.
